# HPV testing alone as a test of cure after treatment with cervical loop excision: a retrospective register-based cohort study

**DOI:** 10.1186/s13027-025-00690-y

**Published:** 2025-08-27

**Authors:** Emma Håstad, Riina Aarnio, Lovisa Bergengren, Matts Olovsson

**Affiliations:** 1 Department of Women’s and Children’s Health, Uppsala University, Akademiska sjukhuset, Uppsala, 751 85 Sweden; 2 Department of Women’s Health, Faculty of Medicine and Health, Örebro University, Örebro, Sweden

**Keywords:** Uterine cervical neoplasms, Papillomavirus infections, Conization, Squamous intraepithelial lesions, Adenocarcinoma *in situ*, Follow-up studies, Retrospective studies

## Abstract

**Background:**

Women treated with cervical loop electrosurgical excision procedure require follow-up to detect residual or recurrent HSIL+, defined as high-grade squamous intraepithelial lesions, adenocarcinoma in situ or cervical cancer. Currently, co-testing with cytology and human papillomavirus (HPV) analysis is usually recommended. This study investigates whether HPV testing alone is comparable to co-testing in detecting HSIL + up to three years after treatment. Recurrence rates of HSIL + are also presented, with follow-up extending up to 18 years.

**Methods:**

This retrospective cohort study included all 3,540 women treated with a cervical excision in Uppsala County between 1 January 2005 and 31 December 2019. Women with cancer identified in the cone biopsy were excluded. The main outcome was HSIL + detected within three years of follow-up. Sensitivity, specificity and negative predictive value were calculated for the 1,938 women who had a co-testing result as part of their test of cure. Thus, the analysis for the main outcome could finally be performed on 1,938 out of the total number of 3,540 women. Additionally, long-term data on recurrence and time to HSIL+, along with a separate analysis of results prior to cervical cancer diagnosis, were collected for the whole cohort of 3,399 women.

**Results:**

The sensitivity and negative predictive value for detecting HSIL + were 69% and 97% for HPV alone, and 74% and 98% for co-testing, respectively. These differences were not statistically significant. Specificity was higher for HPV alone than for co-testing. The negative predictive value of HPV testing for excluding cervical cancer (*n* = 5) within three years was 100%. Recurrence rate of HSIL + in the three-year follow up was 8%, and the total recurrence rate of HSIL + with a mean follow-up of nine years was 10%. Mean time to recurrence was 28 months. None of 19 cervical cancer cases identified in the long-term follow-up had a co-testing result showing negative HPV but positive cytology.

**Conclusions:**

HPV testing alone, as a single test, is comparable to co-testing in detecting HSIL + up to three years after treatment independently of margin status, and demonstrates a higher specificity. Cytology plays a very limited role in the test of cure analysis and could therefore be omitted.

## Background

Cervical cancer is caused by oncogenic human papillomavirus (HPV) [1]. High-grade squamous intraepithelial lesions (HSIL) and adenocarcinoma in situ (AIS) are commonly treated with loop electrosurgical excision procedure (LEEP). Although this treatment is effective in most cases, some women will experience residual or recurrent disease [2, 3]. Risk factors for this include positive margins in the cone biopsy, persistent HPV infection post-treatment, a diagnosis of cervical intraepithelial neoplasia grade 3 (CIN3) and older age. Among these, persistent HPV infection is the most prominent risk factor [2, 4, 5]. Women treated for CIN3 have an increased risk of developing cervical and vaginal cancer later in life [6]. Therefore, post-treatment surveillance is essential to identify women at risk of subsequent high-grade disease. A test of cure (TOC) using both HPV and cytology analysis (co-testing) is recommended six to 12 months after treatment. It is now well-known that HPV has a higher sensitivity than cytology in predicting residual or recurrent disease [7]. Some previous studies have presented a non-significant difference in sensitivity between HPV alone and co-testing as TOC, with a higher specificity for HPV alone [7–9]. Clarke and co-workers concluded that co-testing offers only a small additional risk reduction compared to HPV alone, but at the cost of increased test positivity [8]. A study of Danish women suggested that HPV alone may be acceptable as a TOC when margins of the cone biopsy are clear [10]. The American Society for Colposcopy and Cervical Pathology (ASCCP) guidelines recommend HPV-based testing at six months, and then repeatedly at 18 and 30 months, until the patient has three consecutive HPV negative test results [11]. The Australian recommendations are to have two consecutive negative HPV tests, at 12 and 24 months post-treatment, before returning to routine five-yearly screening [12] In a systematic review from Italy aiming to develop evidence-based guidelines for post-treatment follow-up, a sample analyzed for HPV alone or co-testing at two occasions with the first after six months, is recommended [13]. As far as we are aware, only the UK acknowledges one single negative HPV test, six months post-treatment, to be enough for referring the woman to three-year follow-up [14].

Although the evidence mentioned above supports HPV testing alone as a non-inferior method for follow-up post-treatment, several national guidelines, including the Swedish and other Nordic countries, still recommend co-testing as the preferred TOC [11, 15–17], which is why more studies are needed. There are also remaining questions about the number of negative HPV analyses and the importance of negative margins in the cone biopsy. The main aim of this study was to evaluate whether HPV testing alone, as one single sample, is an acceptable TOC method for predicting the risk of HSIL, AIS or cervical cancer (HSIL+) within three years of follow-up, in a cohort with both positive and negative margins. The secondary aim was to determine the recurrence rate and time to HSIL+, and to analyze the cancer cases separately regarding HPV and cytology results prior to diagnosis across the whole cohort, with follow-up extending up to 18 years.

## Methods

**Fig. 1:**
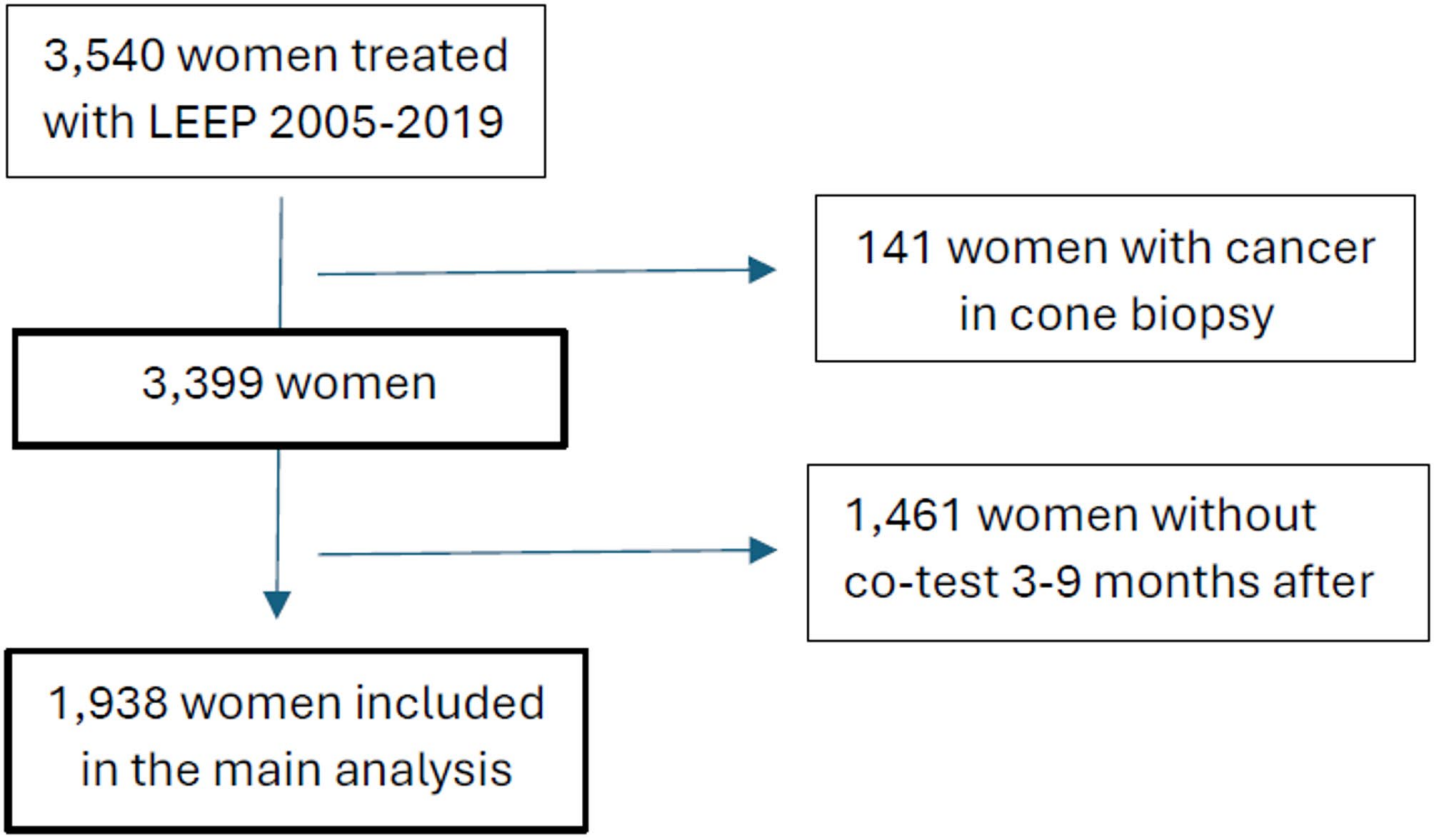
Flowchart of women included in the three-year follow-up analysis after loop electrosurgical excision procedure (LEEP). A number of 3,399 women were included in the study. 1,938 of them had a co-test result as test of cure and were thus included in the main three year-follow up analysis

All women who underwent LEEP in Uppsala County, Sweden, between 1 January 2005 and 31 December 2019 were identified through the hospital medical record system, Cambio COSMIC and were included in the study population. Women with cervical cancer in the cone biopsy at the time of treatment were excluded. Follow-up data were obtained from the County pathology database, SymPathy (TietoEvry AB), which was searched for all available test results relating to cytology, HPV and cervical histopathology using SNOMED codes. The TOC period was defined as three to nine months post-treatment. Women with a co-test result during the TOC period were included in the main analysis, with follow-up ranging from three to 36 months post-treatment (Fig. 1). The proportion of women who were co-tested three to nine months after LEEP was lowest in the beginning of the study period, 11% (2005–2009). From year 2010–2014 there were 47% with a co-test result and in the end of the study period, year 2015–2019, the proportion of co-tested women was 77%.

The Swedish protocol for TOC was at the time a cervical sample analyzed with co-testing after six months. If HPV positive with normal cytology the cervical sampling was repeated after six months, and if still HPV positive the woman was referred for colposcopy. Repeated aberrant cytology or a positive co-test in the first sample also warranted colposcopy [15].

In a separate analysis, here referred to as “HSIL + without specified CIN2”, histopathological diagnoses of confirmed CIN2 (SNOMED codes M74007 and M69717) were excluded from the HSIL + group. As CIN2 and CIN3 were specified as separate entities by the pathologists early in the study period, but later collectively coded as HSIL, some CIN2 cases remain within the “HSIL + without specified CIN2” group.

For the long-term analyses, the entire cohort was followed until women underwent hysterectomy, died, moved to another region, or until the end of data retrieval on 31 December 2023. Data on cone margins were not available in this material. The recurrence rate of HSIL + and time to event were calculated, and an additional analysis was conducted on all cancer cases identified during long-term follow-up, focusing on HPV and cytology results prior to cancer diagnosis.

Three different DNA-based PCR HPV assays were used during the study period. From 2005 to 2009, the Hybrid Capture 2 (HC2; Qiagen, Gaithersburg, MD, USA) method was used. This first-generation comparator test was regarded as a gold standard at the time, and detects 13 high-risk HPV types (16, 18, 31, 33, 35, 39, 45, 51, 52, 56, 58, 59 and 68) [18]. Between 2010 and 2019, samples were collected on FTA (Flinders Technology Associates) cards and analyzed by the HPVIR assay, which is a clinically validated, peer-reviewed PCR test that detects the same 12 HPV types as HC2 [19]. Since January 2020, the HPV assay in use has been BD Onclarity (BD diagnostics, Sparks, MD, USA), which detects six individual HPV types (16, 18, 31, 45, 51 and 52) and eight additional genotypes in three groups (33/58, 35/39/68 and 56/59/66) [20]. From 2005 to 2018, Pap smear was routinely used for cervical cytology and since January 2019, BD SurePath (BD diagnostics, Sparks, MD, USA) liquid-based cytology (LBC) has been implemented. HPV testing was included in the TOC analysis since 2008 in Uppsala County. The laboratory is accredited by the Swedish Board for Accreditation and Conformity Assessment (SWEDAC).

### Statistical analyses

The diagnostic performance of HPV alone vs. co-testing for predicting HSIL + within three years post-treatment was assessed by calculating sensitivity, specificity and negative predictive value (NPV), each with corresponding 95% confidence intervals (CI). Analyses were conducted using R 4.2.1 and Excel 2016.

## Results

A total of 1,938 women were included in the main analysis. The mean age at the time of LEEP was 40 years (SD 11.6, range 19–87 years). The median age was 37 years (IQR 18). A majority of women had a HSIL diagnosis in their cone biopsy histopathology report, 66% (*n* = 1,271). There were 24% cone biopsies with LSIL and 6% with a benign histopathology report (Table 1).

**Table 1:** Histopathological result of the cone biopsy at the time of treatment for women with a co-test result during the test of cure period, after exclusion of women with cancer in the cone biopsy

Histopathological result	number	%
High-grade squamous intraepithelial lesion (HSIL)	1,271	66
Adenocarcinoma in situ (AIS)	45	2
Low-grade squamous intraepithelial lesion (LSIL)	468	24
Benign histopathology	123	6
Other atypia	20	1
Missing data	11	< 1
**Total**	1,938	100

**Fig. 2:**
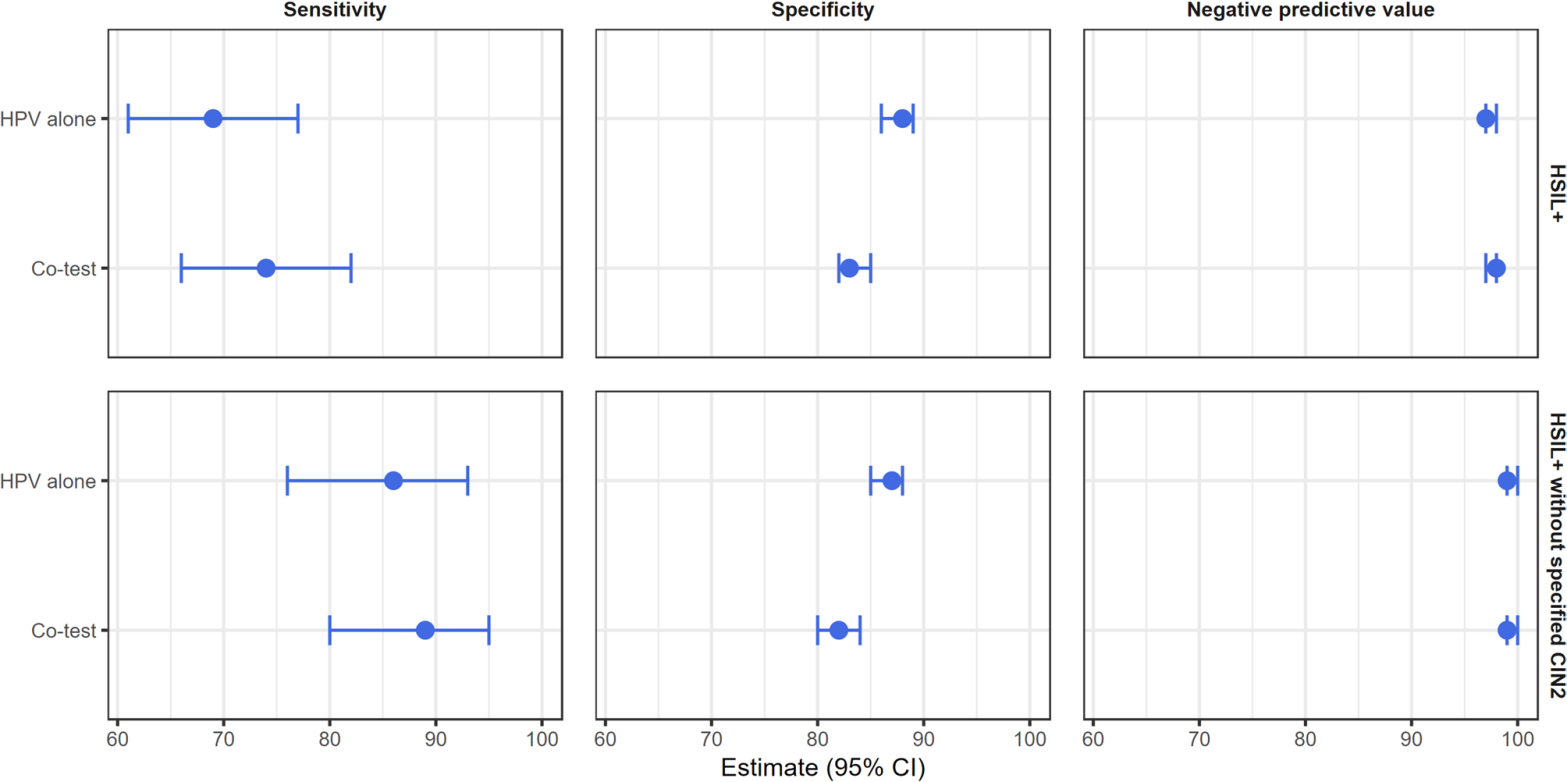
Forest plot of the sensitivity, specificity and negative predictive value of HPV alone and co-testing, respectively, for high grade squamous intraepithelial lesions, adenocarcinoma in situ and cancer (HSIL+) and HSIL + without specified cervical intraepithelial neoplasia grade 2 (CIN2) presented separately

The rate of subsequent HSIL + in the main analysis group up to three years after treatment was 8% (*n* = 158). The rate of HPV positivity during the TOC period three to nine months after treatment was 16% (*n* = 314). The sensitivity and NPV for predicting HSIL + were 69% and 97% for HPV testing alone, and 74% and 98% for co-testing, respectively. When women with a specified CIN2 diagnosis were excluded, the sensitivity and NPV for predicting HSIL + increased to 86% and 99% for HPV testing alone, and 89% and 99% for co-testing, respectively. There was no statistically significant difference in sensitivity or NPV between HPV testing alone and co-testing; however, HPV testing alone demonstrated a higher specificity (Fig. 2).

Among the TOC samples, 314 out of 1,938 were HPV positive. Of 402 women with a positive co-test result, 88 had an aberrant cytology but tested negative for HPV. Seven of these 88 women, corresponding to 0.4% of the total TOC cohort, were diagnosed with HSIL + within three years post-treatment. Of these, five had CIN2 in their cone biopsy histopathology report, received no further treatment and had no subsequent HSIL + diagnoses during long-term follow-up. The remaining two developed HSIL requiring additional treatment.

Of the 1,624 women who were HPV negative in their TOC analysis, nine women developed HSIL + without specified CIN2 diagnoses (See Material and methods) three to 36 months post-treatment, corresponding to 0.55%. Five cases of cervical cancer were detected within the three-year follow-up period. All five had a positive HPV test and aberrant cytology at the time of TOC, resulting in a sensitivity and NPV of 100% for both HPV testing alone and co-testing.

In the long-term follow-up of the whole cohort, comprising a total of 31,688 women years, the mean follow-up duration was 9.3 years (range 4–18 years). The total recurrence rate of HSIL + over the 18-year period was 10% (*n* = 340), with a mean time to HSIL + of 28 months. Nineteen cases of cervical cancer were identified, four adenocarcinomas and 15 squamous cell carcinomas. Seventeen of these women underwent HPV testing at some point before their cancer diagnosis, and none had a positive cytology result coinciding with a negative HPV test.

## Discussion

This study investigated whether HPV testing alone, as a single test, after treatment with LEEP is comparable to co-testing in predicting residual or recurrent HSIL + within three years of follow-up. No significant difference in sensitivity for HSIL + was found. The NPV was similar for both methods, while HPV testing alone demonstrated higher specificity. In the long-term follow-up of up to 18 years, the recurrence rate of HSIL + was 10%, with a mean time to event of 28 months. Concerning the cancer cases (*n* = 17), HPV testing alone detected all women with cancer as effectively as co-testing.

That HPV alone has a comparable sensitivity and higher specificity than co-testing has been shown in previous studies. In an article published almost ten years ago by Asciutto and co-workers, a negative predictive value of 100% for HSIL following a negative HPV test as TOC was presented, however based on a relatively small sample size of 275 women [21]. Bruhn and coworkers concluded that HPV alone might be safe to use as TOC, but only after stratifying for margin status [10]. There is still no international consensus on what TOC analysis should be used, and different countries have different national guidelines. Co-testing is still the most common TOC-analysis used.

In our current data, nine women developed HSIL + when specified CIN2 cases were omitted which corresponds to 0.55% of the total number of women who were HPV-negative in their TOC analysis. This corresponds to the ASCCPs threshold of 0.55% for returning to 5 year-screening [22]. In a Norwegian study, 901 women were HPV negative four to eight months after treatment. Four of the women developed CIN3 + within five years post-treatment with a cumulative risk of CIN3 + within 3 years of 0.33% and within 5 years of 0.45%. Since we did not have distinct CIN2 and CIN3 diagnoses because pathologists coded them together as HSIL later in the study period, by excluding the specimens that were coded as CIN2 specifically, we came as close as possible to a CIN3 + calculation in this material.

A sensitivity of 69% for HPV testing alone and 74% for co-testing was lower than previously reported values, which range from 91 to 94% and up to 96%, respectively [2, 13, 23]. However, when cases with a specified CIN2 diagnosis were excluded, sensitivity for predicting HSIL + increased to 86% for HPV testing alone and 89% for co-testing. The NPV was then 99% for both TOC methods. CIN2 is recognized as a heterogenous diagnosis, with great interobserver variability and poor reproducibility [24–28]. LSIL is generally associated with a productive HPV infection, whereas HSIL implies an infection with neoplastic progression and is thus considered a precancerous lesion. Therefore, the lower anogenital squamous terminology (LAST) recommendations advise the use of immunohistochemistry for p16(INK4a) in CIN2 lesions to better identify potentially oncogenic lesions [26]. However, this method was not routinely implemented in Uppsala County until later in the study period. Immunohistochemistry for p16(INK4a) could potentially have improved diagnostic accuracy [29]. Notably, only two HPV negative cases were later confirmed as HSIL + in histopathology of biopsies.

Our proportion of HSIL in cone biopsies (66%) was similar to another Swedish study with a population from the south of Sweden where 63.7% had a HSIL diagnosis [30]. In our study, 16% of women were HPV positive at the time of TOC, which aligns with findings from other studies reporting 13–21% HPV persistence rates six months post-treatment [31, 32]. No distinction was made between specific type persistence; only the prevalence of any high-risk HPV at the time of TOC was recorded. The recurrence rate of HSIL + was 8% within the three-year follow-up and 10% in the long-term follow-up. Previous studies have reported recurrence rates of 6–7% [2, 10, 33]. HPV testing alone demonstrated 100% sensitivity for predicting cancer and 100% NPV for not missing any cancer cases within the three-year follow-up; however, there were only five cancer cases in this cohort. In the long-term follow-up of all cancer cases, the use of HPV testing alone instead of co-testing as TOC would not have made any difference.

The time frame for TOC was set at three to nine months after LEEP, in accordance with previous studies and the current Swedish recommendations, which advise TOC sampling at four to six months [2, 7, 15]. Most studies included in a meta-analysis evaluated TOC at six months and had follow-up at 24–36 months [8]. We chose three years as the upper limit for follow-up, based on both previous studies and the Swedish guidelines, which recommend a return to routine screening three years after treatment if negative TOC test result.

A possible advantage of using HPV testing alone as a TOC is improved resource efficiency. With co-testing, the number of referrals for colposcopy is higher due to its lower specificity, which increases healthcare costs [9]. Unnecessary colposcopies should also be avoided, as they can be uncomfortable and even painful for women. HPV testing alone could also be implemented as self-sampling, which may improve participation in follow-up in certain groups, as observed in cervical screening programs [34, 35]. Women who do not participate in follow-up have a higher risk of developing cervical cancer, making it crucial to increase coverage [6].

A key strength of this register-based study is the inclusion of a large number (3,399) of participants and a follow-up period of up to 18 years, comprising a total of 31,688 woman-years. The database has high validity with few missing data. Several studies have shown that HPV status is a better predictor of residual or recurrent disease than margin status [2, 36, 37], and that margin status does not influence the performance of HPV testing as a TOC [9, 38]. Although margin status is emphasized in some reports, it is usually not a determining factor in patient management according to national guidelines [12, 14, 15, 39]. Therefore, the lack of margin status in the current study is presumed not to have any significant impact on the results.

Our findings show that the benefit of cytology in the follow-up context is limited and that cytology therefore may be omitted from the TOC routine. The value of repeated HPV testing needs to be further investigated. We propose comprehensive prospective studies to shed further light on this topic and are currently conducting a study evaluating follow-up with self-sampling for HPV testing alone as TOC.

## Conclusions

HPV testing alone, as a single test, is comparable to co-testing for the detection of residual or recurrent HSIL + within three years following treatment with LEEP, independently of margin status. Cytology as a part of TOC has a very limited role in the follow-up routine after LEEP, and could therefore be omitted.

## Data Availability

The datasets used and analyzed during the current study are available from the corresponding author on reasonable request.

## Data availability

No datasets were generated or analysed during the current study.

## Abbreviations

HPV: Human papillomavirus
HSIL: High-grade squamous intraepithelial lesion
AIS: Adenocarcinoma in situ
LEEP: Loop electrosurgical excision procedure
CIN: Cervical intraepithelial neoplasia
NPV: Negative predictive value
TOC: Test of cure
